# Effects of Phytochemicals on Metabolic Diseases and Human Health

**DOI:** 10.3390/nu16244323

**Published:** 2024-12-14

**Authors:** María Ángeles Martín, Sonia Ramos

**Affiliations:** 1 Department of Metabolism and Nutrition, Institute of Food Science and Technology and Nutrition (ICTAN), Consejo Superior de Investigaciones Científicas (CSIC), José Antonio Novais 10, Ciudad Universitaria, 28040 Madrid, Spain; 2 CIBER de Diabetes y Enfermedades Metabólicas Asociadas (CIBERDEM), Instituto de Salud Carlos III (ISCIII), 28029 Madrid, Spain

Metabolic diseases constitute a worldwide health concern because of their increasing prevalence and associated mortality [1,2,3,4]. Actually, pathologies such as type 2 diabetes mellitus, obesity, metabolic syndrome, non-alcoholic fatty liver disease, and cardiovascular diseases are the most common chronic illnesses in almost all countries. Moreover, these diseases are responsible for causing disability in the affected population. All this results in a substantial world economic burden that could be reduced and prevented by lowering the associated risk factors. In this context, metabolic diseases can result from a combination of genetic, physiological, environmental, and behavioral factors. Consequently, a cost-effective solution is to reduce the modifiable behavioral risk factors, with physical activity and a healthy diet being very valuable tools.

Phytochemicals are commonly found in the diet and are abundant in fruits, vegetables, olive oil, legumes, cocoa, and beverages such as tea, coffee, and wine [5]. In fact, because of this wide distribution and their potential effects on health, their consumption may represent a promising protective and therapeutic approach to prevent metabolic diseases and improve health and, therefore, constitutes an important area of research. Notably, phytochemicals have received rising attention for their health-promoting properties in many chronic diseases, including metabolic disorders and their associated complications.

The pathogenesis of the metabolic diseases is very different, but dysregulation of key metabolic signaling pathways (i.e., lipid and glucose homeostasis, insulin pathway, etc.), as well as enhanced oxidative stress and inflammation and alterations in the gut microbiota, are present in all of them [6,7,8,9,10]. In this context, it could be highlighted that the health benefits of the phytochemicals have been related to different biological activities, such as their well-known antioxidant property, but also their anti-diabetic, anti-obesity, and anti-inflammatory actions, among others [11]. It is therefore becoming clear that phytochemicals can modulate different signaling pathways that are responsible for these final actions and, hence, their health benefits.

This Editorial refers to the Special Issue “Effects of Phytochemicals on Metabolic Diseases and Human Health”. The Special Issue highlights the role of oxidative stress, inflammation, and microbiota in the pathophysiology of the different metabolic diseases and comprises thirteen new studies and two reviews. All contributions are listed below:

In this line, Godos et al. have reviewed the clinical studies carried out with flavanols, mainly from cocoa, to expose their impact on vascular health (contribution 1). This review highlights the effects of this family of flavonoids in reducing blood pressure and improving endothelial function, as well as the relevant role that their microbial metabolites play in these final effects by modulating gene expression and protein functions. Likewise, Huang and colleagues (contribution 2) review the potential effects of *Vaccinium* L. on diabetes and microvascular complications, such as nephropathy and retinopathy. In addition, the authors describe recent preclinical and clinical studies focused on the effects of this genus of plants on reducing blood glucose, oxidative stress, and inflammation, which make them excellent candidates for the management of diabetes and diabetic vascular complications, although further studies on the mechanisms of action and larger randomized blinded trials are needed.

Similarly, related to the effects on diabetes, Gallardo-Villanueva et al. report the benefits of a flavanol-rich cocoa–carob blend (CCB) in suppressing islet inflammation by preventing macrophage infiltration into the islets and overproduction of pro-inflammatory cytokines, along with the inactivation of nuclear factor kappa B, which was associated with a prevented beta-cell apoptosis and the loss of beta cells in Zucker Diabetic Fatty (ZDF) rats (contribution 3). In addition, García-Díez and coworkers (contribution 4) demonstrate that this supplemented diet also alleviates damage to the liver and kidneys, key organs for the maintenance of glucose homeostasis, in ZDF rats by ameliorating the oxidative stress, downregulating apoptosis, and improving insulin signaling and glucose homeostasis in both tissues. Interestingly, in both contributions, additive effects were demonstrated when the CCB was combined with metformin, highlighting its potential as an adjuvant therapy to delay the progression of type 2 diabetes. In addition, Zhou and colleagues (contribution 5) proved the mechanism of the hypoglycemic effect of epimedin C, the most important flavonoid of the edible herb *Epimedium*, in diabetic mice. Thus, by using a proteomic approach, the authors demonstrated that epimedin C downregulated key hepatic proteins involved in gluconeogenesis and insulin signaling, such as phosphoenolpyruvate carboxykinase, and upregulated crucial proteins for lipid digestion and degradation (named group XIIB secretory phospholipase A2-like protein, apolipoprotein B-100 [Apob], and cytochrome P450 4A14), resulting in alleviation of lipotoxicity and protection against oxidative stress in the liver of the diabetic animals.

Arterial hypertension constitutes a cause of premature morbidity and mortality. In a randomized, triple-blind controlled trial, Serrano and colleagues (contribution 6) showed that an optimized extract of aged black garlic with low doses of S-allyl cysteine induced an extra-significant reduction in blood pressure in a population of grade I hypertensive patients receiving antihypertensive drug treatment. Importantly, hypercholesterolemia is a central risk factor for atherosclerosis, which is the leading cause of mortality worldwide [4]. In this regard, Langhi et al. explore the mechanism of action of a polyphenol-rich plant extract named Totum-070 in human colonic cells and in hamsters fed a high-fat diet. Totum-070 demonstrated a reduction in various lipid and inflammatory gene expressions and in the intestinal lipid absorption (contribution 7). In addition, this polyphenol-rich extract regulated microbial alpha- and beta-diversity. Indeed, the modulation of both gut microbiota and gut barrier plays a relevant role in metabolic diseases [12]. In this context, Khalil et al. have demonstrated that polyphenolic leave extracts from *Thymbra spicata*, a Mediterranean plant, contributed to maintaining a balanced microbial environment and beneficial metabolites, and it can potentially counteract pathogenic bacterial overgrowth during dysbiosis (contribution 8). Moreover, *T. spicata* extract enhanced intestinal barrier resistance, which is crucial for preserving intestinal integrity and preventing conditions such as leaky gut syndrome. Similarly, Balderas and colleagues (contribution 9) reported an increase in beneficial bacteria in rats fed onion- and apple-enriched diets. Furthermore, a relevant effect of onion and apple intake on major types of microbial-derived molecules, such as short-chain fatty acids (SCFAs) and bile acids (BAs), was demonstrated in obese Zucker rats. Thus, boosted levels of SCFAs and decreased excretion of mainly primary and secondary BAs, as well as increased amounts of taurine- and glycine-conjugated BAs compared to the obese rats, were shown. This differential regulation of bioactive compounds and their metabolites on the microbiota and SCFA and BA formation, as well as their relationship with some diseases, deserves further studies. In this regard, Nederveen et al. in their randomized, double-blind, placebo-controlled clinical trial explore the effects of a novel multi-ingredient supplement on weight loss and body composition in overweight and obese volunteers and demonstrate the effectiveness of this nutritional supplement in reducing weight, fat mass, and markers of liver health and metabolism, such as extracellular vesicle-associated miRNA profiles (contribution 10). At the molecular level, Cierniak and colleagues (contribution 11) show the role of 1,2-dicinnamoyl-*sn*-glycero-3-phosphocholin in increasing the number of mitochondria and the expression levels of genes encoded by mtDNA to ameliorate the insulin resistance in cultured mature 3T3-L1 cells exposed to palmitic acid.

Importantly, the development and progression of liver diseases associated with inappropriate diets is growing worldwide [13], being connected to multiple metabolic diseases. In this regard, Park and coworkers (contribution 12) prove that a homoisoflavonoid isolated from *Portulaca oleracea* named (*E*)-5-hydroxy-7-methoxy-3-(2-hydroxybenzyl)-4-chromanone (HMC) attenuates the hepatic steatosis by decreasing lipid accumulation and triglyceride content in free fatty acid-treated human hepatic HepG2 cells. Mechanistically, HMC promotes fatty acid oxidation via AMPK and PPARα and inhibits key proteins involved in lipid synthesis. Similarly, Donghia et al. in their study cohort report the protective role of lycopene extracts from cooked and fresh tomatoes and conclude that the potent anti-inflammatory and antioxidant properties may serve as a protective factor against the development and progression of steatosis and liver injury (contribution 13). Aiello and coworkers, in a single-arm longitudinal interventional pilot study, also demonstrate that the administration of a dietary supplement based on a liquid residue produced during olive oil extraction has the potential to improve anthropometric, hematological, and metabolic parameters in volunteers at risk of metabolic syndrome (contribution 14).

Anxiety, depression, and cognitive dysfunction are also highly prevalent diseases in our society [14,15]. Wang et al. demonstrate that evodiamine, a major alkaloid from dry unripened fruit *Evodia fructus*, reverses anxiety- and depression-like behavior in rodents (contribution 14). Moreover, evodiamine protected against apoptosis of hippocampal neurons in the post-traumatic stress disorder by modulating the renin–angiotensin pathway.

Overall, this Special Issue provides an update on recent advances in the study of the health benefits of phytochemicals, phytochemical-rich foods, their extracts, and health-promoting benefits in the context of metabolic diseases, as well as revealing novel molecular mechanisms and testing potential novel therapies. This Special Issue highlights the role of oxidative stress, inflammation, and microbiota in the pathophysiology of the different metabolic diseases and the necessity of deepening knowledge about the probable benefits of the phytochemicals against metabolic diseases. In addition, the fact that further in vivo studies are needed before recommending phytochemicals as an alternative/adjuvant approach in the treatment of metabolic diseases is emphasized.
